# Supplementary material for: Proteome‐wide analysis of phospho‐regulated PDZ domain interactions
Source: Mol Syst Biol. 2018 Aug 20;14(8):e8129. doi: 10.15252/msb.20178129 (PMC6100724; doi:10.15252/msb.20178129)
Supplement: Supplementary file 1 — Expanded View Figures PDF [file MSB-14-e8129-s001.pdf]

# Expanded View Figures

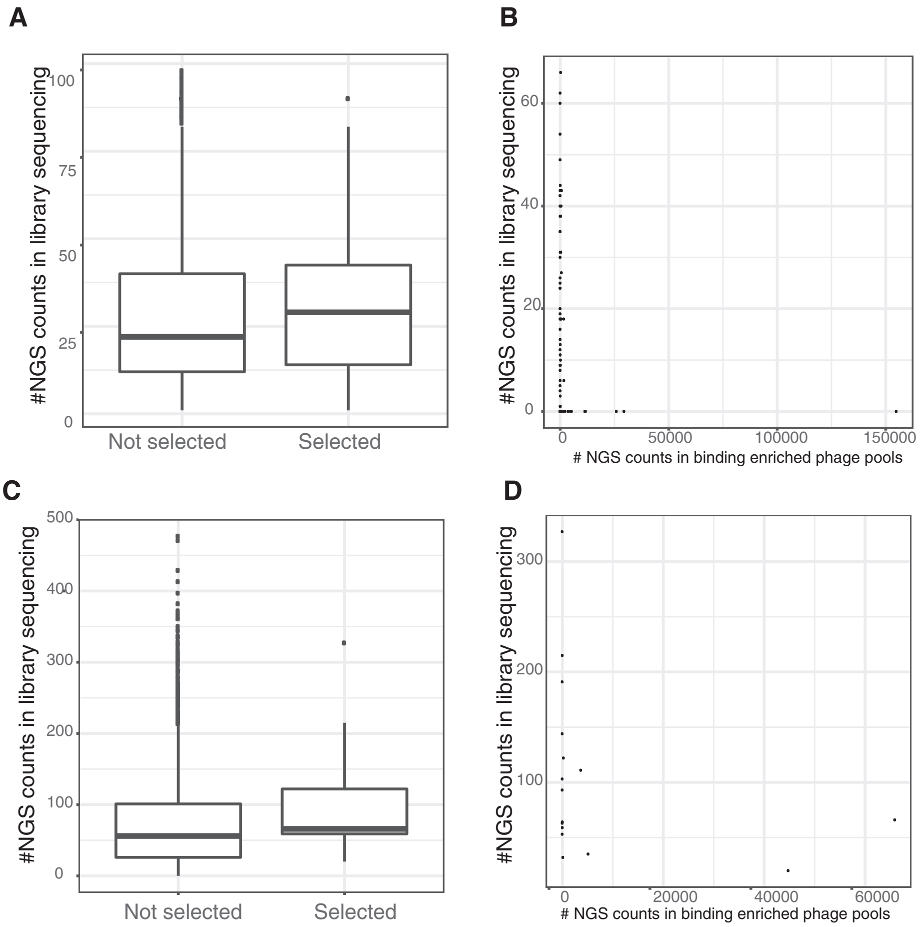


**Figure EV1. Analysis of the phage selection results in comparison with the peptide representation in the naïve phage library.**

A–D Upper panels (A and B): the phosphomimetic ProP-PD experiment, lower panels (C and D): pre-phosphorylation of the wild-type library. Boxplots showing increased probability of a peptide being selected if being well represented in the initial library (*y*-axis: read count in the respective initial library sequencing, peptides with no read support not used). Scatter plots show the read-count after selection in the phage pools (*x*-axis) against the read-counts in the respective library sequencing (*y*-axis).

© 2018 The Authors

© 2018 The Authors

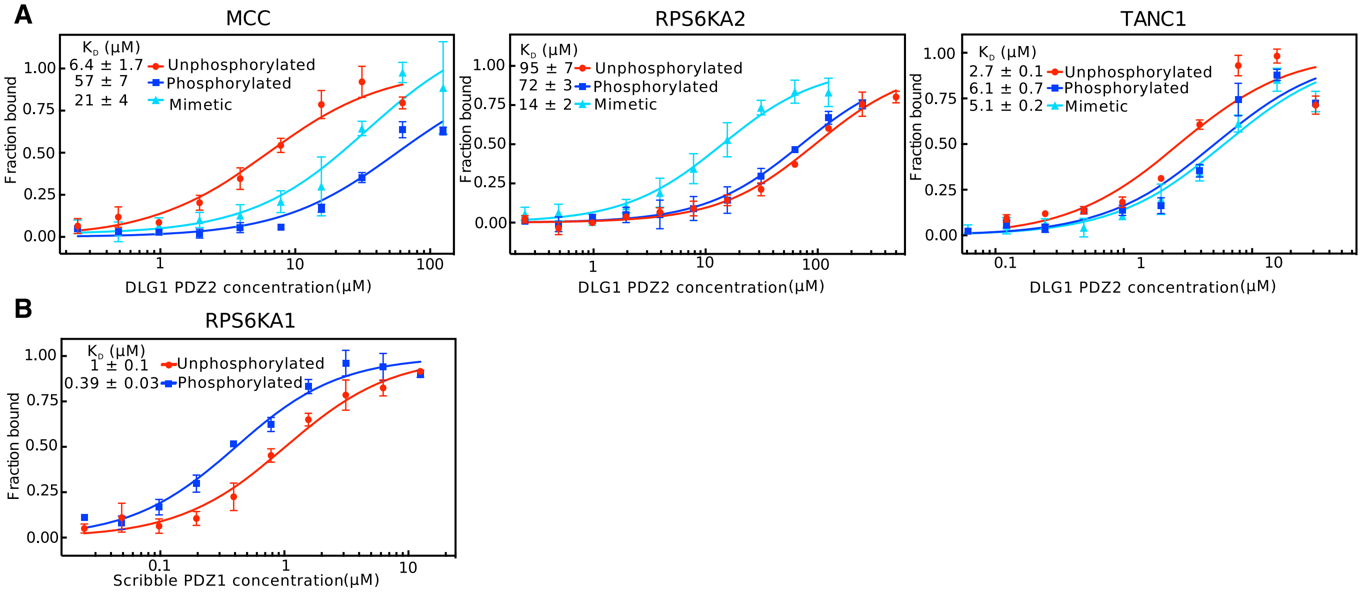


**Figure EV2. Microscale thermophoresis affinity measurements of FITC-labeled peptides and recombinant PDZ domains.**

A fixed peptide concentration (25–50 nM) was titrated with varying concentrations of protein. $K_D$ values were determined using thermophoresis and T Jump signal for data analysis ($n = 3$; error bars represent SD).

A Titration of DLG1 PDZ2 to unphosphorylated, phosphorylated, or phosphomimetic variants of MCC (p-1), RPS6KA2 (p-3), and TANC1(p-6).

B Titration of Scribble PDZ1 to the unphosphorylated or p-3 phosphorylated peptide of RPS6KA1 (VRKLPSTTL-coo-).

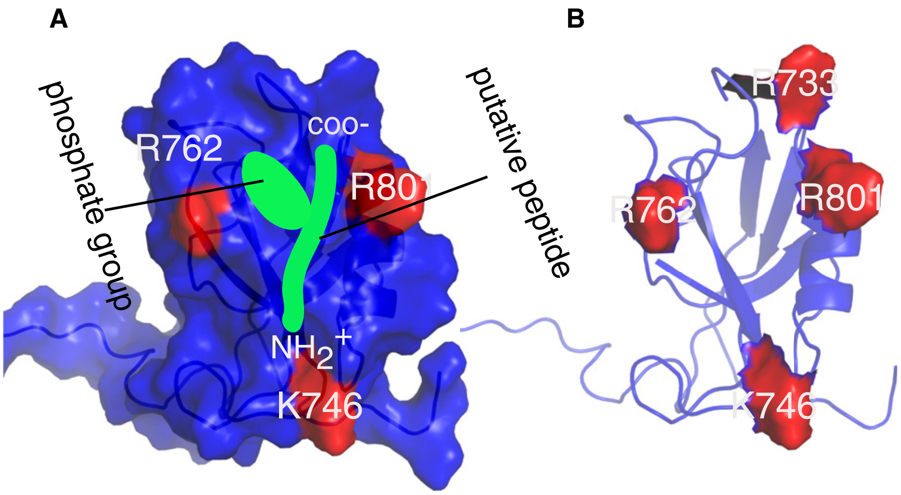


**Figure EV3. Supplementary NMR figures of Scribble PDZ1.**

A Surface representation of Scribble PDZ1 showing charge residues in the vicinity of the putative peptide (green). The carboxylate, amino terminal as well as the phosphate group of the peptide are indicated. Notice the cavity created by the phosphate-group and the C-terminus carboxylate.

B Scribble PDZ1 showing all the positively charged residues surrounding the peptide binding site.


© 2018 The Authors
