# Supplementary material for: Proteome‐wide analysis of phospho‐regulated PDZ domain interactions
Source: Mol Syst Biol. 2018 Aug 20;14(8):e8129. doi: 10.15252/msb.20178129 (PMC6100724; doi:10.15252/msb.20178129)
Supplement: Supplementary file 7 — Table EV6 [file MSB-14-e8129-s007.docx]

Table EV6. NMR structural determination statistics.

| **NMR distance and dihedral restraints** |  |  |  |
| --- | --- | --- | --- |
| **Distance restraints** |  |  |  |
| Total NOEs | 917 |  |  |
| Intra-residue | 310 |  |  |
| Sequential (\|*i* – *j*\| = 1) | 277 |  |  |
| Medium-range (1 < \|*i* – *j*\| < 4) | 138 |  |  |
| Long-range (\|*i* – *j* \| >5) | 192 |  |  |
| Total dihedral angle restraints |  |  |  |
| 3*J*HNα scalar couplings | 72 |  |  |
| 13Cα chemical shifts | 112 |  |  |
| **Structure statistics** |  |  |  |
| Average CYANA target function value (Å2) | 50± 5 |  |  |
| Violations |  |  |  |
| Distance constraints (>0.5 Å) | 8 |  |  |
| Dihedral angle constraints (>5°) | 7 |  |  |
| Deviations from idealized geometry |  |  |  |
| Bond lengths (Å) | 0 |  |  |
| Bond angles (^o^) | 0 |  |  |
| Impropers (^o^) | 0 |  |  |
| ^a^Average pairwise r.m.s. deviation (Å) |  |  |  |
| Backbone | 1.2 ± 0.2 |  |  |
| Heavy atoms | 1.8 ± 0.2 |  |  |
