# Supplementary figures and images for: Proteome‐wide analysis of phospho‐regulated PDZ domain interactions
Source: Mol Syst Biol. 2018 Aug 20;14(8):e8129. doi: 10.15252/msb.20178129 (PMC6100724; doi:10.15252/msb.20178129)

RPS6KA2 GST pulldown

RPS6KA2 WT  $\Delta$ PDZ S-A S-E

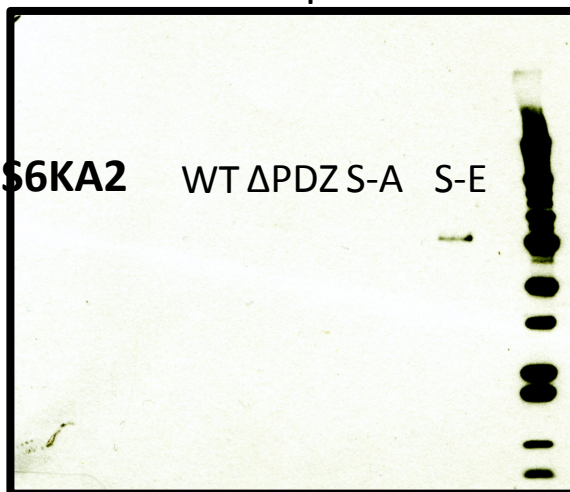

## RPS6KA1 GST pulldown

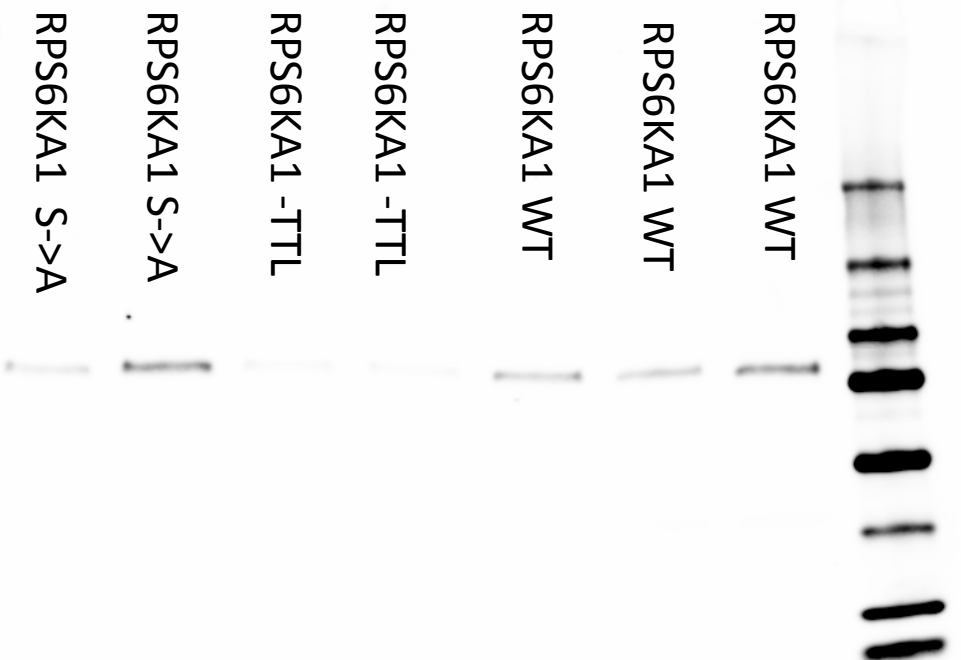

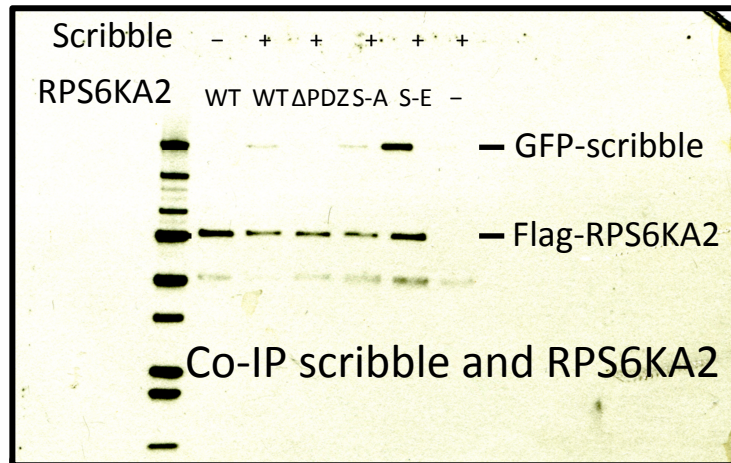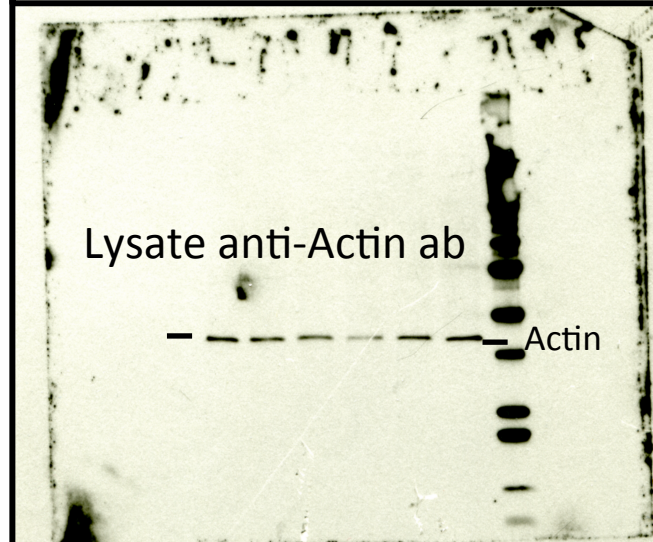

Supplement: Supplementary file 12 — Source Data for Figure 3 [file MSB-14-e8129-s011.pdf]
